# Unusual retrorectal ganglioneuroma: a case report of laparoscopic assisted approach

**DOI:** 10.11604/pamj.2021.38.241.27028

**Published:** 2021-03-07

**Authors:** Ahmed Bouzid, Anis Belhadj, Ahmed Saidani, Ziad Bokal, Fahd Khefacha, Faouzi Chebbi

**Affiliations:** 1 Department of Surgery, Mahmoud El Matri Hospital, University of Medicine Tunis El Manar, Tunis, Tunisia

**Keywords:** Laparoscopy, ganglioneuroma, retrorectal, case report

## Abstract

Ganglioneuromas are benign slow-growing lesions that arise from sympathetic ganglion cells. They are usually found incidentally. Ultrasound and magnetic resonance imaging (MRI), provides only an unspecified diagnosis and it has to be confirmed by pathologic studies. Complete surgical excision is believed to be the curative treatment for symptomatic lesions. In the literature, the pelvic location reported is exceptional. We report a case of laparoscopic assisted excision of a retrorectal presacral ganglioneuroma for 22-year-old female patient.

## Introduction

Ganglioneuromas (GN) are benign slow-growing lesions that arise from sympathetic ganglion cells. They are usually asymptomatic and found incidentally. Surgical excision is believed to be the treatment of choice for symptomatic lesions. In the literature, the pelvic location reported is quite limited. We describe a challenging surgical approach for a huge retrorectal ganglioneuroma.

## Patient and observation

**Figure 1:**
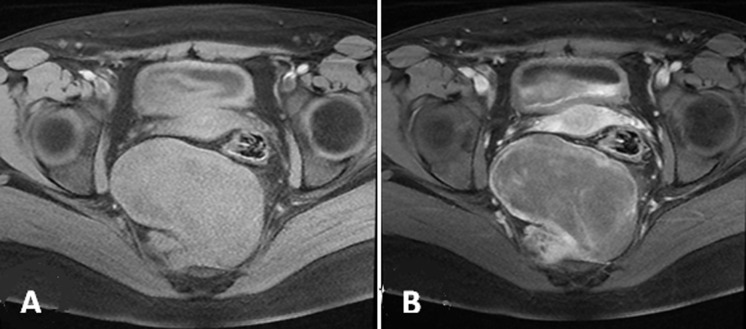
pelvic MRI T1-weighted images FAT-SAT: A) pre gadolinium; B) post gadolinium

**Figure 2:**
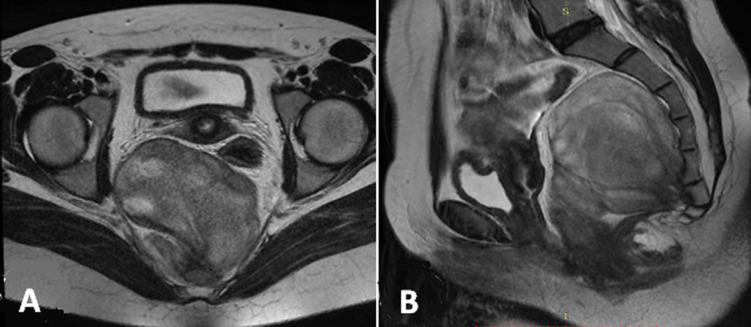
pelvic MRI T2-weighted images: A) axial; B) sagittal

A 22-year-old female with a history of pulmonic stenosis treated with a balloon valvuloplasty presenting lower abdominal pain for last 6 months of visit. She had no history of diarrhea or rectal bleeding. Physical and laboratory examination did not show any pathologic findings except an iron deficiency anemia. Hydatic serology and serous tumor markers (CA12.5, CEA and CA19.9) were at normal ranges. On pelvic ultrasonography (US), we found a hypo echoic presacral lesion without adnexal mass or cyst. Pelvic magnetic resonance imaging (MRI) revealed the presence of an elliptic retrorectal presacral mass measuring 10 x 7 cm. The mass represses the rectosigmoid junction with mass effect and a close contact with the 4^th^ sacred vertebra. T1-weighted images show intermediate signal intensity (Figure 1), whereas T2-weighted images show heterogeneous high signal intensity (Figure 2).

**Figure 3:**
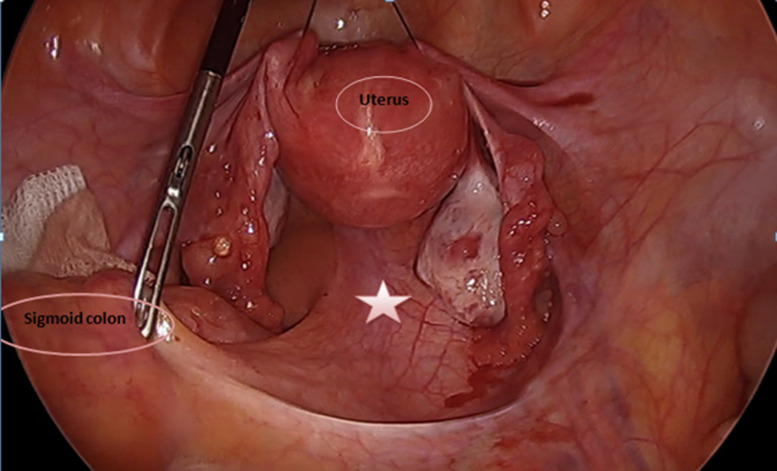
initial view after retracting the sigmoid colon and the uterus

**Figure 4:**
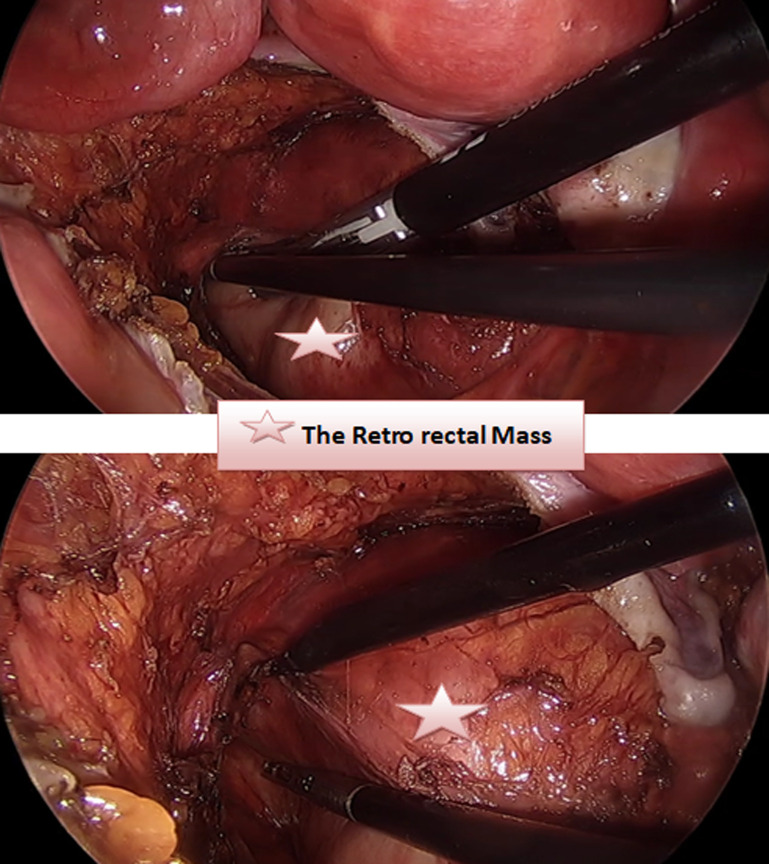
the dissection in the pelvic floor

Prior to excision of the lesion, the patient underwent colonoscopy to ensure the absence of bowel involvement. Initially laparoscopic exploration was performed using three trocars. The sigmoid and the uterus and his adnexa were retracted to expose the tumor in pelvis (Figure 3). The course of the right ureter was seen to be normal, along the right pelvic side wall, anterior and lateral to the mass. The hypogastric nerves and vessels were identified and preserved. After careful identification of L5-S1, the dissection of the promontory starts by cutting the prevertebral parietal peritoneum. Retroperitoneal fat is dissected to allow exposure of the anterior vertebral ligament. Once the tumor is identified, it is carefully dissected to separate the tumor from its attachments to the adjacent structures (Figure 4).

**Figure 5:**
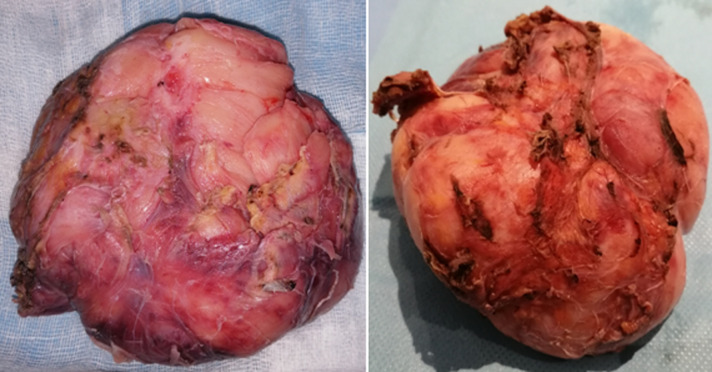
the surgical macroscopic specimen

In the deep pelvic floor, the mass seems to be attached to the posterior wall and the coccyx. Considering the difficulty in handling of the tumor through laparoscopic instruments, we decide to convert to laparotomy. A subtotal resection of the mass was performed via a midline incision. A residual tumor was left behind in an attempt to preserve the nerve root and prevent possible laminectomy for this young girl. The peritoneum was closed with a running suture and a pelvic drainage was performed in order to prevent residual hematoma. The patient had a good postoperative recovery and was discharged home seven days following surgery. The pathology exam confirmed a benign ganglioneuroma with no evidence of neuroblastoma (Figure 5). At one year clinical follow up the patient was symptom free. Further clinical follow up in one year with abdominal computed tomography scan to exclude any radiological recurrence is planned.

## Discussion

According to the International Neuroblastoma Pathology Classification, ganglioneuromas are benign slow-growing lesions that arise from sympathetic ganglion cells predominantly composed of Schwannian stroma with individually distributed maturing/mature ganglion cells [1]. Also, it is revealed that the GNs are often diagnosed in older children. However, there are several reports in the literature in which authors have described malignant transformation of GN into NB has been occasionally reported spontaneously or after radiation therapy [2]. It is located most frequently in the posterior mediastinum, retro peritoneum and adrenal gland. The pelvic location still exceptional and we found few papers involving the pelvic floor [3].

**Diagnosis:** for a considerable time, ganglioneuromas still clinically silent until they reach a large size and becomes symptomatic [4]. The local mass effect can be harmful to the adjacent structures causing an expected complication (constipation, lumbosacral plexus involvement) [4], also, there are functional ganglioneuromas that were found to release peptides such as vasoactive intestinal peptides (VIP), somatostatins and neuropeptide Y (NPY) in the literature [5]. Considering these facts, it would be foolish to ignore the possibility of the hypertension crisis during surgery. So, both the surgeons and the anesthetists should be aware of the blood pressure profile.

**Radiology:** ganglioneuromas is difficult to distinguish from other tumors due to lack of specific image findings. Ultrasound is regarded as the preferred initial screening and diagnostic modality for children with peritoneal tumors because it is non invasive, safe and readily available. But additional diagnostic tools include computed tomography (CT) and magnetic resonance (MR) imaging, which can provide more excellent visualization of tumors and reveal helpful information for surgical approach. The computed tomography cannot confirm the diagnosis but it may be helpful to determinate the extent of the tumor, organ of origin, regional invasion, vascular encasement, adenopathy and calcification. Considering the few findings about retroperitoneal GN and absence of any characteristic radiologic features, the definitive diagnosis is made after a histological study of the surgical piece.

**Management of GN:** the complete surgical excision still the curative treatment [6-8]. Neoadjuvant and adjuvant chemotherapy or radiotherapy is not indicated [9] only if the GN was associated with ganglioneuroblastoma changes [5]. A radical surgery should be the aim of treatment only if it can be achieved without major risk for the patient and/or for adjacent structures. With the knowledge of the tumors biology, some investigators recommend a subtotal resection in difficult cases [7,8]. Recently Decarolis *et al*. has demonstrated surgery does not need to be radical if only minor residuals are left <2 cm [8]. However, surgery should be performed for the following conditions: symptoms resulting from the tumor, encroachment on vertebral foramina, marked growth in size and increased secretory activity of catecholamine [7].

In the most publications the anterior intra peritoneal approach was considered the gold approach in pelvic GN. Other approaches reported in the literature include successful use of the posterior trans sacral approach. This was indicated due to the involvement of sacral nerve roots. In the literature, the laparoscopic approach still under experience and there is no consensus about the place of the mini-invasive surgery in retrorectal mass. Laparoscopy remains possible and even privileged for small well-defined masses without intimate relation to the large vessels [9,10]. It is estimated that new technical development and robotic surgery will offer a new perspective.

**Prognosis and follow up:** the prognosis is good [8] and there are no clear follow up protocols established. In the case of GN recurrence, non-operative management may be justified [8].

## Conclusion

The pelvic ganglioneuroma are rare. The surgical resection is the only curative treatment. In our experience, laparoscopic assisted resection of the pelvic tumor is technically feasible. It is preferred to adopt the surgical approach according to the surgeon experience and each individual patient case, taking account the tumor position, its relationship with the pelvic components and MRI characteristics.
